# BCG-YOLO: an efficient YOLO-based framework for multi-class blood cell detection in microscopic images

**DOI:** 10.3389/fmed.2026.1934762

**Published:** 2026-10-05

**Authors:** Zhu Chen, Yiliang Bao, Yi Liu, Zhongyu Li, Dini Zhang, Meijing Xiao, Yuan Guo, Jinque Luo

**Affiliations:** 1 Hunan Provincial Key Laboratory of the Research and Development of Novel Pharmaceutical Preparations, College of Pharmacy, Changsha Medical University, Changsha, Hunan, China; 2 Hunan Key Laboratory of Biomedical, Nanomaterials and Devices, Hunan University of Technology, Zhuzhou, China; 3 Institute for Future Sciences, University of South China, Changsha, Hunan, China; 4 Department of Transfusion Medicine, Zhuzhou Hospital Affiliated to Xiangya School of Medicine, Central South University, Zhuzhou, Hunan, China

**Keywords:** biomedical image analysis, blood cell detection, lightweight network, microscopic image analysis, small object detection, YOLO11

## Abstract

**Objectives:**

Automated blood cell detection from microscopic images is essential for routine hematological examination and computer-assisted laboratory diagnosis. However, accurate detection remains challenging because of the small size, dense distribution, overlap, and indistinct boundaries of blood cells. This study aimed to develop an efficient deep learning framework for accurate multi-class detection of blood cells in microscopic images.

**Methods:**

We developed BCG-YOLO, an enhanced detector based on YOLO11n that integrates a lightweight backbone network, an improved multi-scale feature fusion module, and a dynamic detection head to improve feature representation and adaptive detection of small and densely distributed blood cells. The proposed model was evaluated on the public BCCD dataset and compared with representative one-stage detectors, transformer-based methods, and real-time object detection models. Ablation studies were conducted to assess the effectiveness of each proposed component. Additional five-fold cross-validation and paired image-level bootstrap analyses were performed for YOLO11n and BCG-YOLO.

**Results:**

On the original fixed test split, BCG-YOLO achieved an mAP@0.5 of 95.3%, an mAP@0.5:0.95 of 67.6%, and a recall of 89.8% while requiring only 3.80 GFLOPs. Under the common training and evaluation protocol used in this study, BCG-YOLO achieved the best overall detection performance among all evaluated models. Ablation experiments further demonstrated the effectiveness of the proposed modules, confirming consistent improvements in overall detection performance and computational efficiency.

**Conclusions:**

BCG-YOLO provided a favorable balance between detection accuracy and computational cost in the internal evaluation on BCCD. These findings support its feasibility for microscopic blood-cell detection, while independent external validation is required to establish generalizability across laboratories, staining protocols, and imaging systems.

## 1 Introduction

Blood cell detection is a fundamental component of routine hematological analysis and plays an important role in the diagnosis of anemia, leukemia, infectious diseases, and other hematological disorders. Conventional microscopic examination relies heavily on experienced laboratory personnel and is labor-intensive, time-consuming, and susceptible to observer variability, particularly when processing large numbers of samples. Recent reviews indicate that artificial intelligence applied to peripheral blood films and hematological morphology can improve analytical efficiency and standardization, although expert validation and well-characterized clinical data remain essential (1, 2).

Deep learning has significantly advanced medical image analysis, with convolutional neural networks (CNNs) supporting classification, segmentation, object detection, and quantitative analysis (3). In blood-cell microscopy, recent lightweight one-stage detectors have further demonstrated the feasibility of efficient automated cell localization (4). Compared with image classification, blood cell detection requires simultaneous localization and classification of multiple cell types, making it substantially more challenging. Platelets are extremely small, red blood cells are densely distributed and frequently overlap, and different blood cell types exhibit diverse morphologies, textures, and staining patterns. Imaging artifacts and background noise further complicate feature extraction. Recent YOLO-based blood-cell detectors therefore increasingly combine lightweight backbones, multi-scale fusion, attention, and boundary-sensitive feature enhancement to address accuracy–efficiency trade-offs in these complex scenes (5, 6).

Recent studies have increasingly adopted deep learning for automated blood cell analysis. Segmentation networks such as U-Net provide accurate pixel-level representation (7), whereas object detection models are more suitable for simultaneously predicting blood cell categories and locations (8, 9). The availability of public blood cell datasets has further promoted the development of deep learning algorithms for microscopic image analysis (10–14). Recent improvements, including lightweight architectures, multi-scale feature fusion, attention mechanisms, and optimized training strategies, have substantially enhanced blood cell detection performance (15–19).

Despite these advances, several challenges remain. First, repeated downsampling weakens shallow features and edge information, leading to missed detection of tiny targets such as platelets (20, 21). Second, conventional feature fusion networks mainly enhance semantic information but remain insufficient for representing local structures and blurred boundaries in densely distributed and overlapping blood cells (21, 22). Third, most existing detection heads perform static feature aggregation and cannot dynamically adapt to targets of different scales and spatial distributions. Meanwhile, increasing model complexity to improve accuracy is undesirable for practical deployment in intelligent clinical laboratory systems.

To address these challenges, we propose BCG-YOLO, an efficient blood cell detector based on YOLO11n. The proposed model introduces three complementary improvements. First, a Lightweight Ghost-Based Backbone Network (LGBNet) is adopted to improve feature extraction while reducing computational redundancy. Second, Poly-scale Convolution (PSConv) is incorporated into the neck to enhance the representation of multi-scale local structures, overlapping regions, and blurred boundaries. Third, Dynamic Head (DyHead) replaces the original detection head to dynamically recalibrate features across the scale, spatial, and task dimensions, thereby improving the detection of small and densely distributed blood cells. Experimental results on the public BCCD dataset demonstrate a favorable balance between detection accuracy and computational cost, providing an initial proof of concept for automated microscopic blood-cell detection. The design is organized as a task-to-module mapping rather than an unstructured accumulation of general detection components. LGBNet targets the loss of shallow detail and redundant computation, PSConv targets fixed-receptive-field limitations in overlapping and weak-boundary regions, and DyHead targets static feature aggregation across heterogeneous scales and spatial distributions. Table 1 summarizes this task-specific rationale and the supporting evidence available in the present study.

**Table 1:** Task-specific design rationale and level of supporting evidence.

Blood-smear challenge	Baseline limitation	BCG-YOLO response	Location	Evidence in this study
Tiny platelets and weak local contrast	Detail loss during repeated downsampling	HGStem, GhostHG, and DWConv preserve efficient shallow representations	Backbone	Single-module ablation; class-wise and qualitative evidence for the complete model
Dense and overlapping cells with blurred boundaries	Fixed receptive fields provide limited local structural modeling	PSConv introduces poly-scale sampling and boundary-sensitive fusion	Neck	Single-module ablation and qualitative overlap examples
Heterogeneous target scales and spatial distributions	Static aggregation in the original detection head	DyHead performs scale-, spatial-, and task-aware recalibration	Head	Single-module ablation; class-wise PR curves and confusion matrices for the complete model
Resource-constrained deployment	Accuracy improvements may substantially increase computation	Ghost operations and depthwise convolution reduce redundant computation	Backbone	GFLOPs, parameter-count, and measured FPS comparisons

## 2 Related work

### 2.1 YOLO-based blood cell detection

Recent blood-cell detectors increasingly combine lightweight convolution, multi-scale fusion, attention, and loss reweighting. Han et al. developed a lightweight one-stage WBC detector and evaluated it across Raabin-WBC, BCCD, and LISC (4). EB-YOLO (5) and LWF-YOLO (6) further emphasize the balance between accuracy and deployment efficiency. TW-YOLO evaluated multi-scale fusion on several blood-cell datasets (15), while subsequent studies explored YOLOv11 optimization (16), two-stage detection and classification (17), Swin-based multi-scale attention (18), and loss reweighting for imbalanced blood-cell detection (19). These studies demonstrate rapid progress, but their datasets, label spaces, data splits, augmentation pipelines, and reporting practices differ substantially; consequently, numerical results across papers are not directly comparable without a shared evaluation protocol.

### 2.2 Transformer-based and real-time medical detectors

Transformer-based detectors provide an alternative to conventional one-stage architectures by using set prediction and global feature interaction. DETR and Deformable DETR established the principal end-to-end detection framework (28, 29), while RT-DETR and Gold-YOLO extended real-time detection through efficient feature interaction (30, 31). Recent medical-object-detection studies have also shown that DETR-family performance is sensitive to optimizer, pretraining, schedule length, and model-specific hyperparameters (34). Accordingly, the comparisons in this manuscript should be interpreted as controlled results under a common protocol rather than estimates of the independently optimized upper-bound performance of every architecture.

## 3 Materials and methods

### 3.1 Overall architecture of BCG-YOLO

BCG-YOLO is a multi-class blood cell detector constructed from YOLO11n. It retains the basic backbone–neck–head structure of a single-stage detector but introduces coordinated improvements for dense small objects, blurred boundaries, and local overlaps in microscopic blood cell images. The overall architecture is shown in Figure 1.

**Figure 1:**
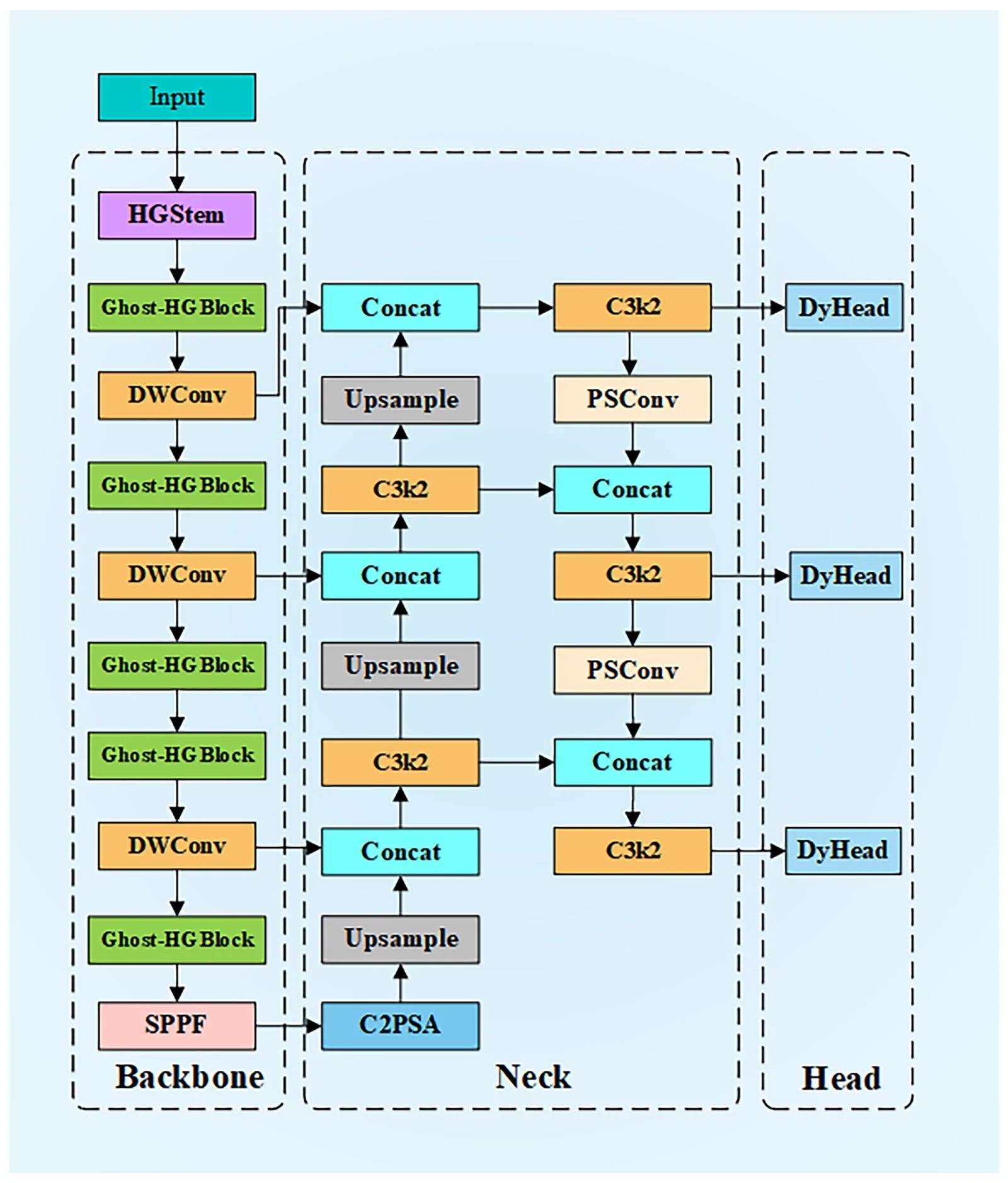
Overall architecture of BCG-YOLO.

The input image is first processed by LGBNet to extract multi-scale hierarchical features. Compared with the original YOLO11n backbone, LGBNet combines HGStem, GhostHG, depthwise separable convolution (DWConv), and spatial pyramid pooling-fast (SPPF) modules to build a more efficient feature-extraction pathway while preserving shallow edge and texture information. The neck then transfers features bidirectionally through top-down and bottom-up fusion paths. PSConv modules are introduced at key positions to strengthen the representation of local structures, boundary regions, and overlapping cells at different scales. Finally, DyHead replaces the original detection head and applies dynamic attention across the scale, spatial, and task dimensions. These modifications are not isolated additions: LGBNet improves the efficiency of basic feature extraction, PSConv strengthens boundary-sensitive fusion, and DyHead dynamically optimizes features before classification and bounding-box regression.

For a 640 × 640 input, LGBNet produces P3, P4, and P5 tensors of 64 × 80 × 80, 128 × 40 × 40, and 256 × 20 × 20 at strides 8, 16, and 32, respectively. PSConv is inserted after selected C3k2 fusion blocks in the top-down and bottom-up neck paths. Before DyHead, 1 × 1 convolutions project the three feature levels to 128 channels, producing tensors of 128 × 80 × 80, 128 × 40 × 40, and 128 × 20 × 20. DyHead performs scale-aware, spatial-aware, and task-aware recalibration without changing the spatial resolutions. For three blood-cell classes and reg_max = 16, each prediction level contains 67 output channels (4 × 16 + 3), and the three levels collectively contain 8,400 candidate locations. Table 2 summarizes the module locations, tensor transformations, and comparison with the original YOLO11n structure.

**Table 2:** Structural comparison and tensor flow between YOLO11n and BCG-YOLO.

Stage	YOLO11n	BCG-YOLO	Output tensor/scale	Purpose of modification
Stem/backbone	Original YOLO11n convolutional backbone	HGStem, GhostHG blocks, DWConv, and SPPF	64 × 80 × 80; 128 × 40 × 40; 256 × 20 × 20	Preserve shallow detail while reducing redundant computation
Top-down neck	Upsample, Concat, and C3k2 fusion	Retained fusion path with PSConv after selected C3k2 blocks	128 × 80 × 80; 128 × 40 × 40	Improve local and boundary-sensitive fusion
Bottom-up neck	Concat and C3k2 aggregation	Retained aggregation path with PSConv at selected fusion positions	128 × 40 × 40; 128 × 20 × 20	Model overlap and blurred boundaries at multiple scales
Detection head	Original YOLO11n Detect head	1 × 1 channel projections followed by DyHead at three feature levels	67 × 80 × 80; 67 × 40 × 40; 67 × 20 × 20	Adaptive scale-, spatial-, and task-aware recalibration

### 3.2 Lightweight ghost-based backbone network

The backbone is responsible for extracting low-level textures, local morphological patterns, and high-level semantic information. In microscopic blood cell images, small sizes, dense arrangements, adhesive boundaries, and subtle inter-class differences make shallow-detail preservation particularly important. Excessive loss of detail during downsampling can cause missed detections and confusion between similar categories. Standard convolution can also impose a substantial computational burden in deeper networks. To improve practical efficiency without sacrificing detection performance, the original YOLO11n backbone is replaced with a Lightweight Ghost-Based Backbone Network (LGBNet).

LGBNet consists primarily of HGStem, GhostHG blocks, DWConv, and SPPF. GhostHG combines the Ghost feature-generation strategy with the hierarchical aggregation mechanism of HGBlock. HGStem comprises five convolutional layers and one max-pooling operation, while HGBlock reorganizes features through multi-branch cascading and concatenation. The overall backbone and GhostHG block are shown in Figure 2.

**Figure 2:**
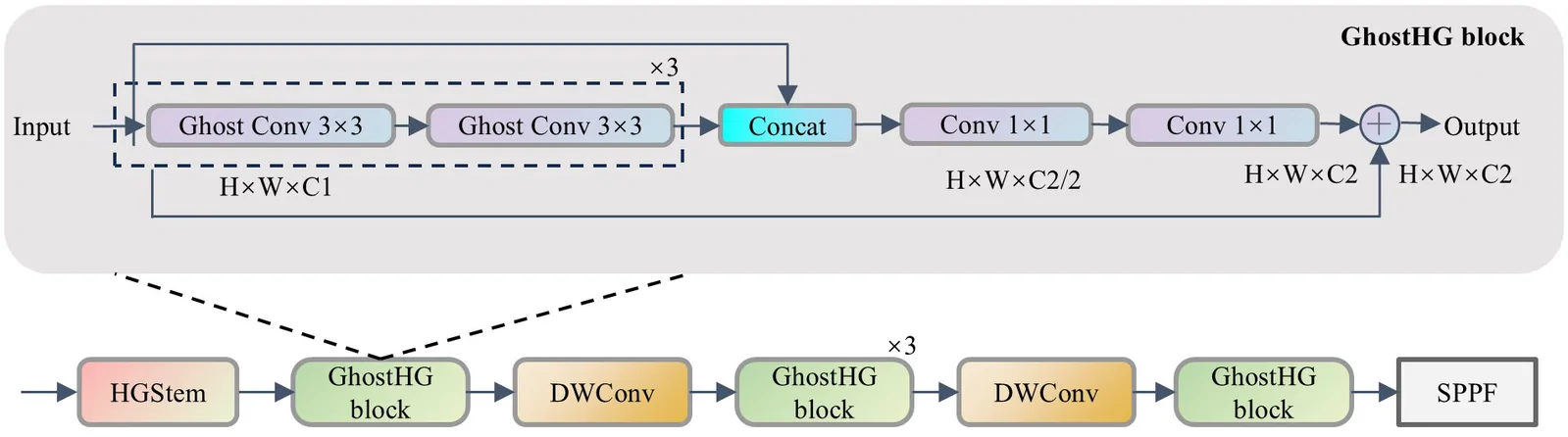
Structure of the LGBNet backbone and GhostHG module.

HGStem is introduced at the input of the backbone. Multiple small convolutional kernels and pooling operations progressively downsample the image. Compared with a conventional large-stride convolution, this approach retains more edge information and local textures while reducing spatial resolution. This is important for detecting small or weak-contrast blood cell targets.

During backbone feature extraction, GhostHG replaces conventional stacked blocks to reduce redundant computation. GhostNet first generates a set of intrinsic feature maps using a small number of standard convolutions and then produces additional Ghost features through inexpensive linear transformations (23). The Ghost feature-generation process is defined in Equations 1 and 2:

$$Y^{\prime }\;=\;X\;*\;{\;f}^{\prime }$$ (1)

$$y_{i,j}=\Phi_{i,j}({y^{\prime }}_{i})$$ (2)

where *X* denotes the input feature map, *f*′ denotes the primary convolution kernel, and *Φ* denotes an inexpensive linear transformation. In BCG-YOLO, GhostHG uses this low-cost feature generation within hierarchical aggregation, while DWConv separates spatial filtering from channel mixing. Their role here is computational: the backbone is redesigned to reduce redundant operations while retaining the shallow representations needed by the downstream three-scale neck. A structural comparison between standard convolution and depthwise separable convolution is shown in Figure 3.

**Figure 3:**
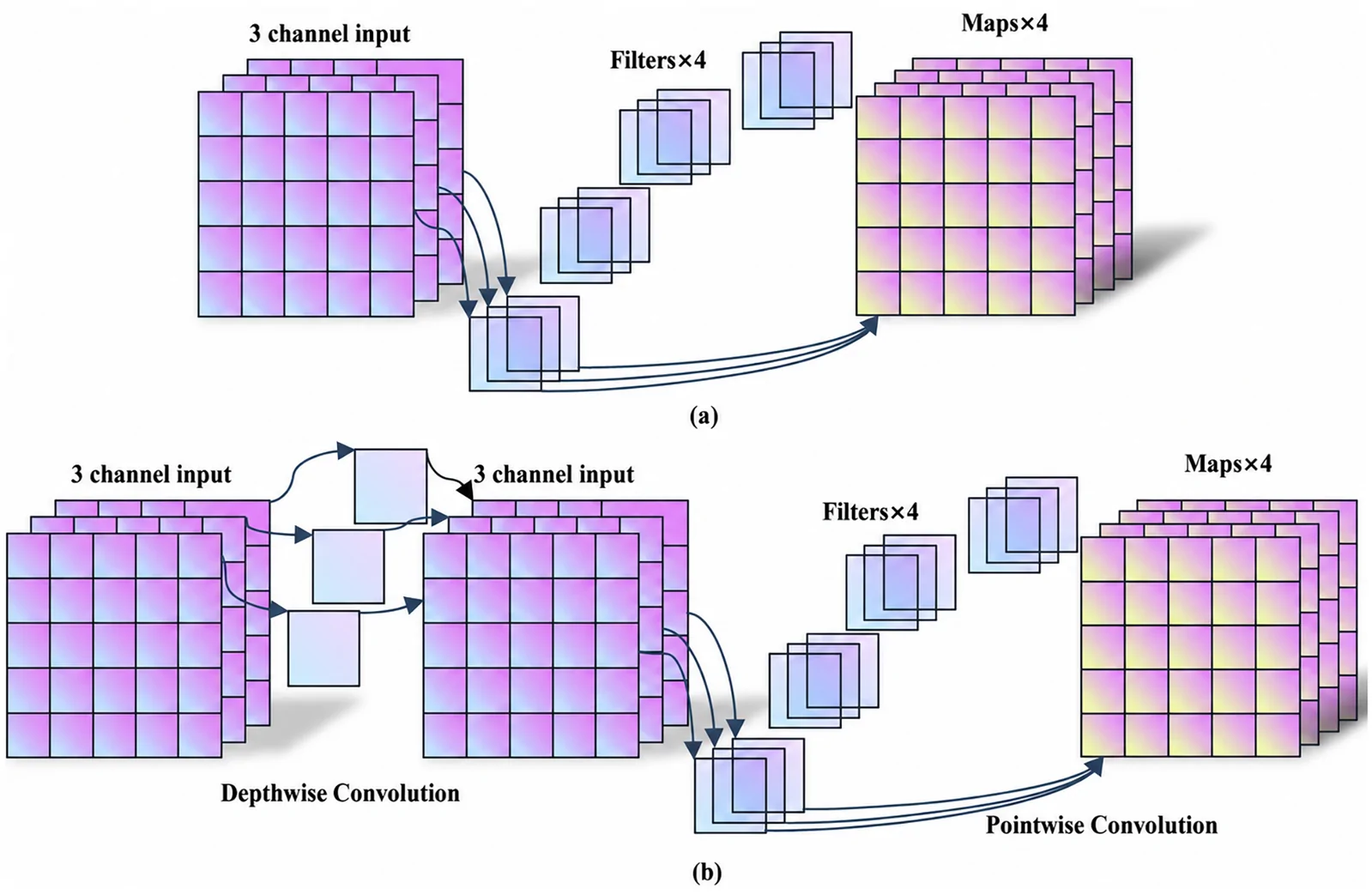
Comparison of standard convolution and depthwise separable convolution (DWConv).

The computational complexity of standard convolution is given in Equation 3:

$$C_{\mathrm{std}}=D_{K}^{2}\cdot M\cdot N\cdot D_{F}^{2}$$ (3)

The computational complexity of depthwise separable convolution is given in Equation 4:

$$C_{dw}=D_{K}^{2}\cdot M\cdot D_{F}^{2}+M\cdot N\cdot D_{F}^{2}$$ (4)

DWConv therefore decomposes convolution into channel-wise and pointwise operations, substantially reducing computational requirements while maintaining effective feature extraction (24). Finally, SPPF is introduced at the end of the backbone to enlarge the receptive field through repeated pooling and to aggregate local and global information at low computational cost.

### 3.3 Poly-scale convolution module

The neck integrates features across scales and directly affects detection in dense or complex scenes. In microscopic images, overlapping targets, blurred boundaries, and low local contrast make it difficult for standard convolution with fixed receptive fields to preserve both fine-grained boundary information and multi-scale local structures. PSConv is introduced into the neck to improve feature fusion (25). By incorporating multi-scale receptive fields and sparse connectivity within a single convolutional layer, PSConv strengthens the representation of local structures and boundary regions at different scales. Partial standard convolutions in the neck are replaced with PSConv to improve discrimination in overlapping-cell regions and along blurred boundaries. The module is shown in Figure 4.

**Figure 4:**
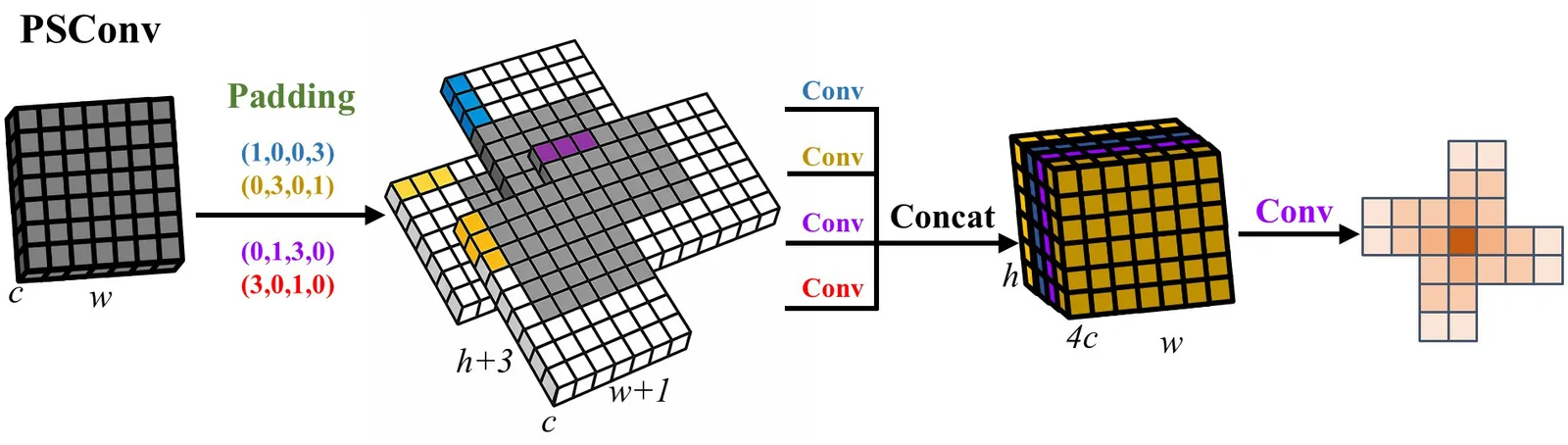
Detailed structure of the PSConv module.

PSConv combines responses from differently sampled local regions within one compact operation. In BCG-YOLO, it is inserted at selected neck fusion positions shown in Figure 1 and Table 2, where it supplements the fixed-receptive-field C3k2 pathway with poly-scale local sampling. The intended role is to improve feature fusion around cell contours, partially overlapping regions, and weak-texture boundaries without directly enlarging every convolutional kernel.

A standard convolution is formulated in Equation 5:

$$Y(p)=\sum_{k\in R}W(k)\cdot X(p+k)$$ (5)

where *X* is the input feature map, *W* is the convolution kernel weight, *R* is the receptive-field region, and *p* denotes a pixel location. This formulation lacks explicit modeling of local structures and boundaries at different scales. PSConv extends this operation into a combination of sparse sampling regions or multi-scale branches, as defined in Equation 6:

$$Y(p)=\sum_{d\in D}\sum_{k\in R_{d}}W_{d}(k)\cdot X(p+k)$$ (6)

where *D* denotes the set of directions, *R_d_* denotes the sampling region for direction d, and *W_d_* is the corresponding convolution weight. PSConv models feature variations in different directions and thereby strengthens structural and boundary perception. Sparse sampling and shared weights limit the growth in parameter count, allowing richer spatial information to be acquired at lower cost than directly enlarging kernels or stacking additional convolutional layers.

### 3.4 Dynamic detection head

BCG-YOLO replaces the standard YOLO11n detection head with DyHead so that the final prediction features can adapt to the visual variability of blood cells. The three incoming pyramid levels contain different amounts of spatial detail and semantic information, and their usefulness changes with target size, position, and prediction task. Before classification and bounding-box regression, DyHead therefore reweights the features along the scale, spatial, and task dimensions. The resulting head is illustrated in Figure 5.

**Figure 5:**
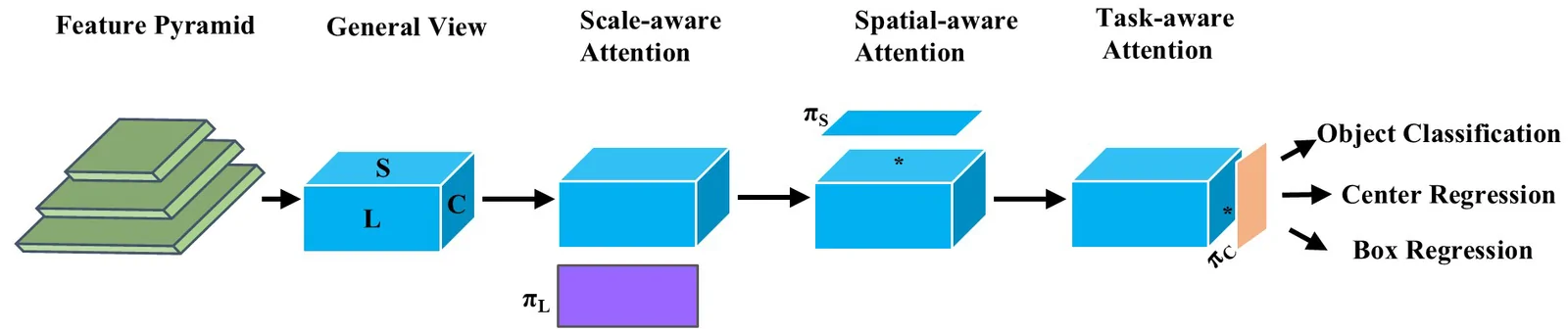
Architecture of the dynamic head (dyHead).

Let the multi-level output of the neck be arranged as $F\in R^{L\times S\times C}$, where L is the number of feature levels, S is the number of spatial positions, and C is the number of channels. Applying one attention operation over this complete tensor would be computationally expensive. DyHead instead associates the three dimensions with complementary attention mechanisms, and its overall transformation is defined in Equation 7:

W(F)=π(F)⋅F (7)

For tractable feature recalibration, the operator is applied successively to the feature-level, spatial, and task dimensions:

$$W(F)=\pi_{C}(\pi_{S}(\pi_{L}(F)\cdot F)\cdot F)\cdot F$$ (8)

The three operators in Equation (8) correspond to scale-aware, spatial-aware, and task-aware attention, respectively. Scale-aware attention determines the relative contribution of each pyramid level, allowing features associated with different target sizes to be weighted before prediction (27). The scale-aware attention mechanism is formulated in Equation 9:

$$\pi_{L}(F)\cdot F=\sigma \left(\;f\left(\frac{1}{SC}\sum_{S,C}F\right)\right)\cdot F$$ (9)

The scale-aware branch first summarizes each feature level over its spatial and channel dimensions, transforms the result with the 1 × 1 convolution *f*(·), and applies the hard-sigmoid function *σ*(·) to obtain a level weight. This operation retains the contribution of high-resolution features when small cells are present instead of treating all pyramid levels identically. Spatial-aware attention then uses sparse sampling and cross-level aggregation to select informative positions (26):

$$\pi_{S}(F)\cdot F=\frac{1}{L}\sum_{l=1}^{L}\sum_{k=1}^{K}w_{l,k}\cdot F(p_{k}+\Delta p_{k})\cdot \Delta m_{k}$$ (10)

In Equation (10), *K* is the number of sparse sampling points, *pκ* + Δ*pκ* is a learned sampling location, Δ*mκ* is its importance coefficient, and *w1*,*κ* is the aggregation weight across feature levels and sampling points. Gathering responses at selected locations directs the spatial branch toward cell edges, local textures, and regions between adjacent cells. The task-aware branch subsequently adjusts channel responses for classification and localization, after which the recalibrated features are passed to the prediction layers.

### 3.5 Evaluation metrics

Detection performance was evaluated with mAP@0.5, mAP@0.5:0.95, and recall, while GFLOPs and parameter count were used to describe computational cost and model size. At a selected intersection-over-union (IoU) threshold, a prediction is counted as a true positive when its class is correct and its bounding box matches a ground-truth object. Unmatched predictions and missed ground-truth objects contribute false positives and false negatives, respectively.

For each blood-cell class, average precision (AP) is calculated using Equation 11 by integrating precision over the recall range:

$$\mathrm{AP}=\;\int_{0}^{1}\mathrm{Precision}(\mathrm{Recall})d(\mathrm{Recall})$$ (11)

The reported mean average precision is calculated as the arithmetic mean of the class AP values, as shown in Equation 12:

$$\mathrm{mAP}=\frac{1}{N}\sum_{i=1}^{N}AP_{i}$$ (12)

Here, AP*i* denotes the AP of class i and *N* is the number of target classes. For mAP@0.5, AP is calculated at an IoU threshold of 0.5. For mAP@0.5:0.95, AP values are averaged over thresholds from 0.5 to 0.95 in steps of 0.05, providing a stricter assessment of localization quality. GFLOPs represent the floating-point operations required for one forward pass, whereas parameter count reflects model size.

Each of the three blood-cell classes contributes one AP value to mAP, so the aggregate mAP is not directly weighted by the number of RBC, WBC, or platelet instances. Accordingly, mAP@0.5 and mAP@0.5:0.95 are reported together with recall. Table 3 additionally provides class-specific precision, recall, AP@0.5, and AP@0.5:0.95 for the two principal models.

**Table 3:** Class-wise detection performance of YOLO11n and BCG-YOLO on the BCCD test set.

Model	Class	Precision/%	Recall/%	AP@0.5/%	AP@0.5:0.95/%
YOLO11n	RBC	92.5	89.4	94.7	66.5
	WBC	90.8	87.6	93.2	65.1
	Platelet	85.9	82.2	87.2	53.8
BCG-YOLO	RBC	95.2	92.1	97.1	72.0
	WBC	93.7	90.4	96.1	69.1
	Platelet	91.1	86.9	92.7	61.7

### 3.6 Statistical analysis

An image-level paired bootstrap analysis was performed to quantify uncertainty in detection performance on the original 36-image BCCD test set. A total of 5,000 bootstrap resamples were generated by sampling 36 images with replacement in each iteration. All ground-truth annotations and predictions associated with each sampled image were retained, and repeatedly sampled images were treated as separate copies during evaluation. Identical resampling indices were used for YOLO11n and BCG-YOLO to preserve the paired comparison.

For each resample, mAP@0.5, mAP@0.5:0.95, and recall were recalculated using the original evaluation settings. The 95% confidence intervals were obtained from the 2.5th and 97.5th percentiles of the bootstrap distributions. Performance differences were calculated as BCG-YOLO minus YOLO11n within each resample, and their confidence intervals were derived from the paired difference distributions. The analysis used the same model checkpoints and test images as the comparison reported in Table 4.

**Table 4:** Aggregate performance comparison under the common BCCD protocol.

Model	mAP@0.5/%	mAP@0.5:0.95/%	Recall/%	GFLOPs	Params/M	FPS
YOLOv8n	93.4	65.8	88.0	8.10	3.01	284.1
YOLOv9e	94.1	66.9	88.6	192.50	57.3	87.9
YOLOv10n	92.8	65.0	87.4	6.70	**2** **.** **30**	293.3
YOLO11n	91.7	61.8	86.4	6.30	2.58	297.6
Gold-YOLO-N	92.3	63.9	86.9	12.10	5.60	223.7
RT-DETR-R50	90.8	63.1	85.7	136.00	41.90	103.8
RT-DETR-HGNetv2-L	92.1	64.5	86.8	110.00	62.80	101.2
Deformable DETR	89.6	61.2	84.9	173.00	18.60	82.4
BCG-YOLO	**95** **.** **3**	**67** **.** **6**	**89** **.** **8**	**3** **.** **80**	3.72	**305**.**8**

Bold values indicate the best result in each metric column: the highest mAP@0.5, mAP@0.5:0.95, recall, and FPS, and the lowest GFLOPs and parameter count.

## 4 Results

### 4.1 Dataset

The publicly available Blood Cell Count and Detection (BCCD) dataset was used to evaluate BCG-YOLO (33). It contains 364 peripheral blood-smear images at a native resolution of 640 × 480 pixels and 4,888 bounding-box annotations: 4,155 RBCs (85.01%), 372 WBCs (7.61%), and 361 platelets (7.39%). The repository distributes the images and annotations in PASCAL VOC format under the MIT License (33). Only publicly released images and annotations were used; no new participants, specimens, clinical records, or directly identifiable patient information were collected. Platelets are often approximately 10 × 10 pixels, while overlap, blurred boundaries, and staining artifacts are common. The images were randomly divided once at the image level using a predefined 7:2:1 ratio, yielding 255 training images, 73 validation images, and 36 test images after integer rounding. This fixed split was used for the original baseline comparisons, ablation experiments, and matched-seed evaluations. Additional five-fold cross-validation was performed for YOLO11n and BCG-YOLO, as described in Section 4.5. Independent external validation was not performed. Representative samples are shown in Figure 6.

**Figure 6:**
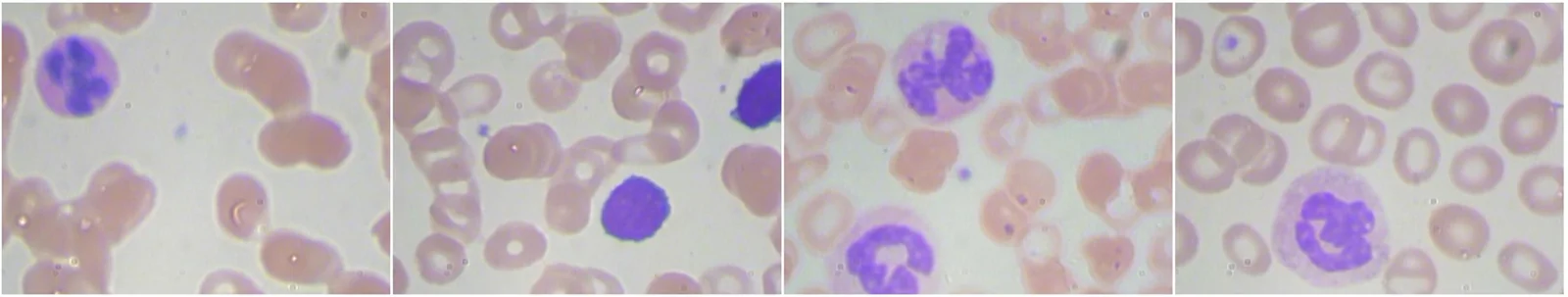
Representative images from the BCCD dataset.

### 4.2 Experimental setup

All experiments were conducted on a cloud server under a unified Linux environment. Training and inference were implemented in PyTorch with CUDA acceleration, and stochastic gradient descent (SGD) was used for optimization. The YOLO11n baseline and BCG-YOLO were implemented with the official YOLO11 model definition in Ultralytics 8.3.0; YOLOv8n and YOLOv10n were run in the same Ultralytics environment, whereas YOLOv9e, Gold-YOLO-N, RT-DETR, and Deformable DETR followed their official public implementations. Official COCO-pretrained weights were used where available. For BCG-YOLO, compatible YOLO11n parameters were transferred, while the newly introduced LGBNet, PSConv, DyHead, and projection layers used the framework's default initialization. The common hyperparameters were selected from the official default training recipes and fixed before test evaluation; the test set was not used for hyperparameter selection. All compared models were evaluated on the same data split, input resolution, hardware platform, and common training protocol. This design controls several experimental variables but does not constitute model-specific hyperparameter optimization, particularly for Transformer-based detectors that may prefer different optimizers and schedules. The comparison should therefore be interpreted within this common protocol. The recorded environment and training settings are summarized in Table 5. The input resolution was 640 × 640 pixels, the batch size was 16, and training lasted 200 epochs. The initial learning rate was 0.1 and was reduced to 0.01 using cosine scheduling. Data augmentation included HSV perturbation (hue 0.015, saturation 0.7, value 0.4), random translation (0.1), scaling (0.5), horizontal flipping (0.5), Mosaic (1.0; disabled during the final 20 epochs), RandAugment, random erasing, MixUp, and Copy-Paste. For each run, the checkpoint with the highest validation mAP@0.5:0.95 during the 200 epochs was selected and evaluated once on the held-out test set. YOLO11n and BCG-YOLO were additionally trained with five matched random seeds; the image split and hyperparameters were held constant, and only seed-dependent initialization and training order varied. The other baseline and ablation configurations were evaluated once.

**Table 5:** Experimental environment, training protocol, and validation scope.

Experimental parameter	Value
GPU	NVIDIA GeForce RTX 4090
CUDA cores	16,384
GPU memory	24 GB GDDR6X
Operating system	Ubuntu 22.04
Integrated development environment	Visual Studio Code
Python version	Python 3.10
PyTorch version	PyTorch 2.1.0
CUDA version	CUDA 12.1
Input resolution	640 × 640 pixels
Batch size	16
Training epochs	200
Optimizer	SGD
Learning rate	0.1 initially; cosine decay to 0.01
Data split	Fixed: 255/73/36 images; five-fold CV
Evaluation repetitions	Fixed split: 5 seeds for YOLO11n/BCG-YOLO; 1 run for others
Cross-validation	Five-fold; YOLO11n and BCG-YOLO
Model-specific tuning	Common controlled protocol
Implementation sources	Ultralytics 8.3.0 (YOLO11n/YOLOv8n/YOLOv10n); official repositories (other baselines)
Pretraining and initialization	Official COCO weights; compatible YOLO11n weights transferred; new BCG-YOLO layers default-initialized
Checkpoint selection	Best validation mAP@0.5:0.95 within 200 epochs; one final test evaluation
Statistical analysis	Paired image-level bootstrap; 5,000 resamples

### 4.3 Comparison study

To evaluate BCG-YOLO under the common protocol described above, representative object detectors were compared on the same BCCD split. The models included YOLOv8n, YOLOv9e, YOLOv10n (32), YOLO11n, Gold-YOLO-N, RT-DETR variants, and Deformable DETR. Because the models were not independently tuned, the results in Table 4 represent controlled protocol-specific comparisons rather than each architecture's optimized upper-bound performance.

The YOLO11n baseline used the official Ultralytics 8.3.0 model definition and the public COCO-pretrained yolo11n.pt checkpoint. It was trained for the full 200 epochs on the same 255-image training set and monitored on the same 73-image validation set as BCG-YOLO. The checkpoint with the highest validation mAP@0.5:0.95, rather than the final-epoch checkpoint, was evaluated once on the 36-image test set. Under this controlled protocol, BCG-YOLO achieved the highest aggregate metrics in Table 4, with an mAP@0.5 of 95.3%, mAP@0.5:0.95 of 67.6%, recall of 89.8%, and 3.80 GFLOPs. Relative to the selected YOLO11n checkpoint, the differences were 3.6 percentage points in mAP@0.5, 5.8 percentage points in mAP@0.5:0.95, and 3.4 percentage points in recall. YOLO11n nevertheless obtained a lower mAP@0.5 than YOLOv8n, indicating sensitivity to the common implementation and optimization settings. Table 4 reports the principal point estimates for all architectures. The five matched-seed results for YOLO11n and BCG-YOLO are presented separately in Table 6, whereas the remaining baselines were evaluated once. Thus, the repeated-run evidence supports the stability of the improvement over YOLO11n, while the broader cross-architecture comparison remains specific to the shared protocol.

**Table 6:** Repeated-run performance of YOLO11n and BCG-YOLO across five random seeds.

Model	Runs	mAP@0.5/%	mAP@0.5:0.95/%	Recall/%
YOLO11n	5	91.70 ± 0.35	61.80 ± 0.56	86.40 ± 0.51
BCG-YOLO	5	95.30 ± 0.32	67.60 ± 0.41	89.80 ± 0.56
Mean improvement	—	+3.60	+5.80	+3.40

Table 3 presents the class-wise detection performance of YOLO11n and BCG-YOLO. BCG-YOLO achieved better results for all three blood-cell classes. For RBCs, AP@0.5 and AP@0.5:0.95 increased from 94.7% and 66.5% to 97.1% and 72.0%, respectively. For WBCs, AP@0.5 increased from 93.2% to 96.1%, while AP@0.5:0.95 increased from 65.1% to 69.1%. The most substantial improvement was observed for platelets: recall increased from 82.2% to 86.9%, AP@0.5 increased from 87.2% to 92.7%, and AP@0.5:0.95 increased from 53.8% to 61.7%. Combined with the overall mAP results reported in Table 4, these findings demonstrate that the performance improvement of BCG-YOLO was observed across all three blood-cell classes rather than being driven solely by the majority RBC class.

Figure 7 further compares the class-wise detection characteristics of YOLO11n and BCG-YOLO. BCG-YOLO produced higher precision–recall curves for all three blood-cell classes. Compared with YOLO11n, AP@0.5 increased from 94.7% to 97.1% for RBCs, from 93.2% to 96.1% for WBCs, and from 87.2% to 92.7% for platelets. The largest improvement of 5.5 percentage points was therefore observed for platelets. The diagonal values of the normalized confusion matrices increased from 0.894, 0.876, and 0.822 with YOLO11n to 0.921, 0.904, and 0.869 with BCG-YOLO for RBCs, WBCs, and platelets, respectively. Correspondingly, the background false-negative proportions decreased for all three classes. These results indicate that BCG-YOLO improves not only the aggregate mAP but also the detection of the relatively underrepresented platelet class.

**Figure 7:**
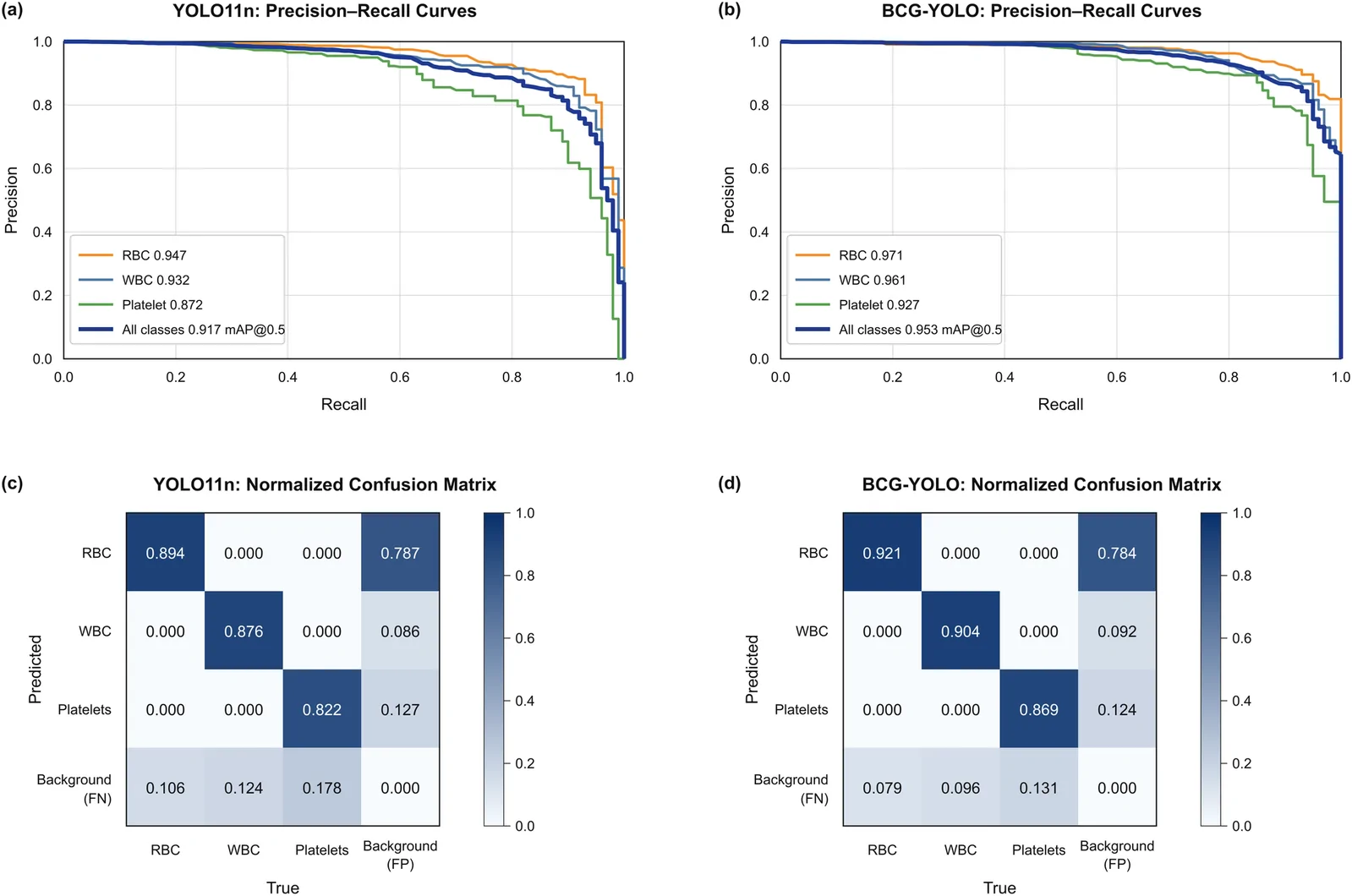
Class-wise precision–recall curves and normalized confusion matrices of YOLO11n and BCG-YOLO on the BCCD test set: **(a)** precision–recall curves of YOLO11n; **(b)** precision–recall curves of BCG-YOLO; **(c)** normalized confusion matrix of YOLO11n; and **(d)** normalized confusion matrix of BCG-YOLO. The precision–recall curves were calculated at an IoU threshold of 0.5. Background FP denotes unmatched predictions, whereas background FN denotes unmatched ground-truth objects.

Table 6 summarizes the results of the five repeated runs. BCG-YOLO achieved an average mAP@0.5 of 95.30 ± 0.32%, an mAP@0.5:0.95 of 67.60 ± 0.41%, and a recall of 89.80 ± 0.56%. In comparison, YOLO11n achieved 91.70 ± 0.35%, 61.80 ± 0.56%, and 86.40 ± 0.51%, respectively. BCG-YOLO outperformed YOLO11n under all five matched random seeds. The mean improvements of 3.60 percentage points in mAP@0.5, 5.80 percentage points in mAP@0.5:0.95, and 3.40 percentage points in recall were substantially larger than the observed run-to-run standard deviations, indicating that the performance gains were consistent across random initializations under the fixed data split.

Figure 8 presents a qualitative comparison of the detection results produced by YOLO11n and BCG-YOLO. Panel (a) shows the original images, while panels (b) and (c) display the predictions generated by YOLO11n and BCG-YOLO, respectively. In the first example, BCG-YOLO successfully identifies red blood cells near the image boundary that are missed by YOLO11n. In the second example, BCG-YOLO provides more accurate detection of overlapping blood cells in the central and right-hand regions. In the third example, BCG-YOLO correctly detects a small platelet located near the top of the image, further demonstrating its effectiveness in detecting small blood cell targets.

**Figure 8:**
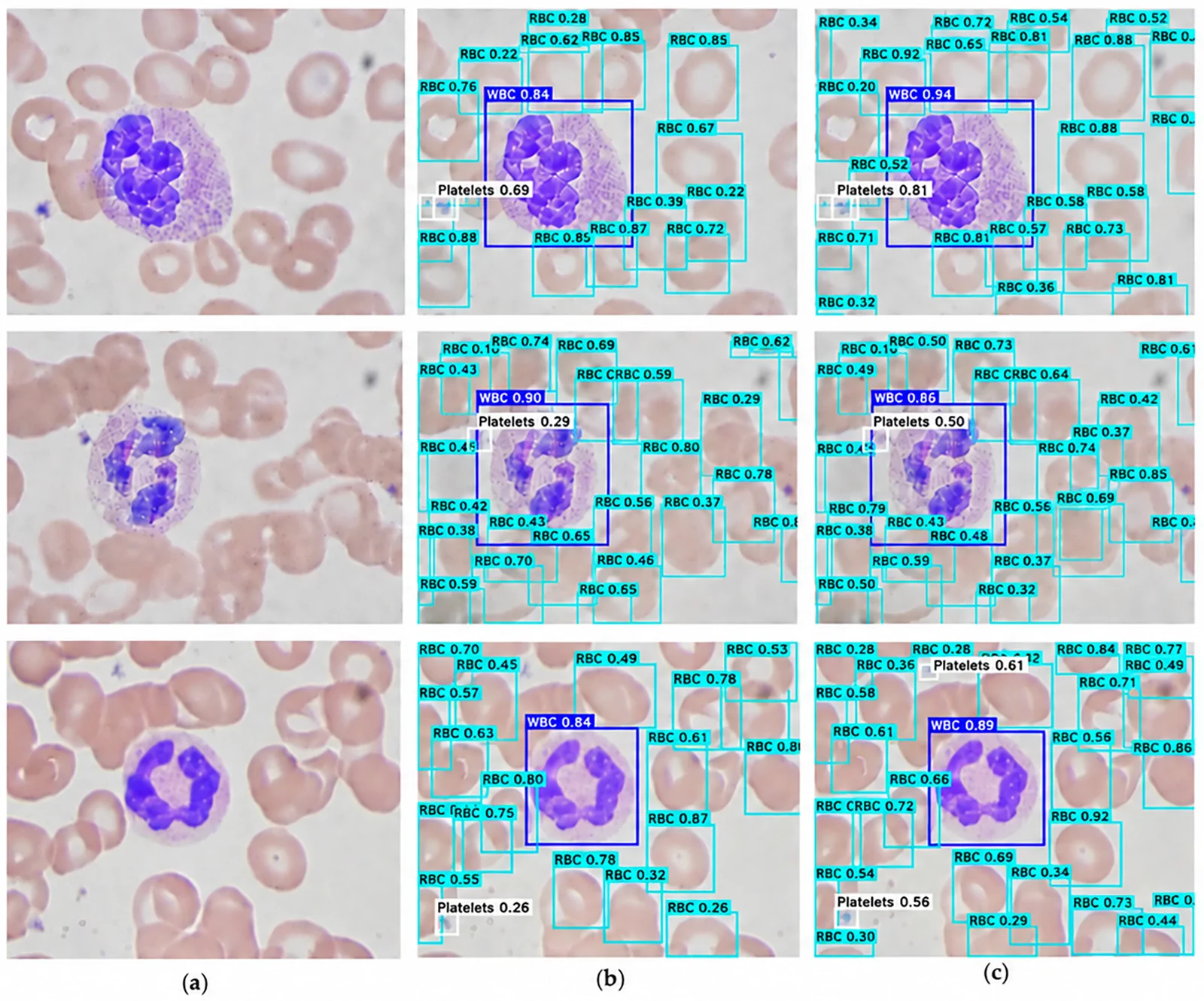
Visual comparison of detection outputs produced by YOLO11n and BCG-YOLO: **(a)** original images; **(b)** YOLO11n predictions; and **(c)** BCG-YOLO predictions.

To further characterize the resource requirements of BCG-YOLO, Table 7 compares its computational cost, parameter count, and weight storage with those of YOLO11n. Weight storage is reported for FP32 and FP16 representations.

**Table 7:** Computational cost and model weight storage of YOLO11n and BCG-YOLO.

Model	Parameters (M)	GFLOPs	FP32 weight storage (MB)	FP16 weight storage (MB)
YOLO11n	2.58	6.30	10.32	5.16
BCG-YOLO	3.72	3.80	14.88	7.44

BCG-YOLO required 3.80 GFLOPs, representing a 39.7% reduction compared with YOLO11n. Together with the higher detection accuracy reported in Table 4, this result demonstrates an improved balance between detection performance and computational cost. The parameter count increased from 2.58 M to 3.72 M. The corresponding weight storage was 14.88 MB in FP32 and 7.44 MB in FP16 for BCG-YOLO, compared with 10.32 MB and 5.16  MB, respectively, for YOLO11n.

### 4.4 Ablation study

To comprehensively evaluate the individual contributions and interactions of the proposed modules, we conducted a complete factorial ablation study covering all eight configurations of LGBNet, PSConv, and DyHead relative to the YOLO11n baseline. In addition to the individual-module configurations, all three two-module combinations and the complete BCG-YOLO model were evaluated under the same training and evaluation protocol. The results are summarized in Table 8.

**Table 8:** Ablation study of the proposed BCG-YOLO framework.

YOLO11n	LGBNet	PSConv	DyHead	mAP@0.5/%	mAP@0.5:0.95/%	GFLOPs	Params/M
√				91.7	61.8	6.30	2.58
√	√			93.1	63.6	**3** **.** **20**	**2** **.** **36**
√		√		92.8	63.2	6.54	2.91
√			√	93.0	63.9	6.88	3.24
√	√	√		94.4	65.9	3.48	3.05
√	√		√	94.2	65.7	3.78	3.02
√		√	√	94.5	66.3	7.12	3.57
√	√	√	√	**95** **.** **3**	**67** **.** **6**	3.80	3.72

Bold values indicate the best result in each metric column: the highest mAP@0.5 and mAP@0.5:0.95, and the lowest GFLOPs and parameter count.

In the reported experiments, LGBNet, PSConv, and DyHead individually improved the aggregate detection performance relative to the YOLO11n baseline. LGBNet additionally reduced the computational cost from 6.30 to 3.20 GFLOPs and the parameter count from 2.58 M to 2.36 M. Among the two-module configurations, LGBNet + PSConv achieved an mAP@0.5 of 94.4% and an mAP@0.5:0.95 of 65.9%, LGBNet + DyHead achieved 94.2% and 65.7%, respectively, and PSConv + DyHead achieved 94.5% and 66.3%, respectively. The complete BCG-YOLO model achieved the best aggregate performance, with an mAP@0.5 of 95.3% and an mAP@0.5:0.95 of 67.6%, exceeding all single-module and two-module configurations. These results are consistent with complementary contributions from the three modules under the experimental protocol used in this study.

### 4.5 Five-fold cross-validation results

To assess the sensitivity of model performance to data partitioning, five-fold cross-validation was conducted on the BCCD dataset. The 364 images were divided into five non-overlapping folds containing approximately 72–73 images each. In each iteration, one fold was reserved for testing, while the remaining four folds were used for training and validation. Each image therefore served as a test sample exactly once. YOLO11n and BCG-YOLO were evaluated using identical data partitions. Table 9 summarizes the results as the mean ± standard deviation across the five test folds.

**Table 9:** Five-fold cross-validation performance of YOLO11n and BCG-YOLO on the BCCD dataset.

Model	Folds	mAP@0.5/%	mAP@0.5:0.95/%	Recall/%
YOLO11n	5	91.70 ± 0.71	61.9 ± 1.12	86.34 ± 0.92
BCG-YOLO	5	94.42 ± 0.72	66.80 ± 1.22	89.10 ± 0.87
Mean difference	—	**+2.72**	**+4.90**	**+2.76**

Bold values indicate the mean performance improvements of BCG-YOLO over YOLO11n.

BCG-YOLO achieved a mean mAP@0.5 of 94.42 ± 0.72%, a mean mAP@0.5:0.95 of 66.80 ± 1.22%, and a mean recall of 89.10 ± 0.87%. The corresponding values for YOLO11n were 91.70 ± 0.71%, 61.90 ± 1.12%, and 86.34 ± 0.92%, respectively. BCG-YOLO therefore exceeded YOLO11n by an average of 2.72 percentage points in mAP@0.5, 4.90 percentage points in mAP@0.5:0.95, and 2.76 percentage points in recall. The direction of these differences agrees with the original fixed-split comparison, providing additional evidence of higher average detection performance for BCG-YOLO under different BCCD data partitions.

### 4.6 Uncertainty analysis

Table 10 reports the detection metrics and their image-level bootstrap 95% confidence intervals for YOLO11n and BCG-YOLO on the original BCCD test set. The paired analysis quantifies uncertainty in the performance differences while accounting for the evaluation of both models on the same test images.

**Table 10:** Detection performance and paired differences with image-level bootstrap 95% confidence intervals on the original BCCD test set.

Metric	YOLO11n (95% CI)	BCG-YOLO (95% CI)	Paired difference (95% CI)
mAP@0.5 (%)	91.70 [88.40, 94.50]	95.30 [92.60, 97.20]	+3.60 [+0.70, +6.50]
mAP@0.5:0.95 (%)	61.80 [57.10, 66.20]	67.60 [63.00, 71.90]	+5.80 [+1.60, +10.10]
Recall (%)	86.40 [81.30, 90.80]	89.80 [85.20, 93.50]	+3.40 [−0.60, +7.30]

BCG-YOLO achieved an mAP@0.5 of 95.30% (95% CI: 92.60–97.20%), compared with 91.70% (95% CI: 88.40–94.50%) for YOLO11n. The paired difference was 3.60 percentage points (95% CI: 0.70–6.50). For mAP@0.5:0.95, BCG-YOLO achieved 67.60% (95% CI: 63.00–71.90%), exceeding YOLO11n by 5.80 percentage points (95% CI: 1.60–10.10). The paired confidence intervals for both mAP metrics excluded zero, providing statistical support for improved detection performance under the evaluated conditions. Recall increased from 86.40% for YOLO11n to 89.80% for BCG-YOLO. The paired difference was 3.40 percentage points, with a 95% confidence interval of −0.60 to 7.30 percentage points.

## 5 Discussion

This study proposed BCG-YOLO, an improved model for multi-class blood-cell detection in microscopic images. By incorporating a lightweight backbone, a multi-scale feature-fusion module, and a dynamic detection head, the proposed model enhances the representation of small, densely distributed blood cells with blurred boundaries. Under the unified experimental protocol and BCCD data split adopted in this study, BCG-YOLO achieved an mAP@0.5 of 95.3%, an mAP@0.5:0.95 of 67.6%, and a recall of 89.8%, with a computational cost of only 3.80 GFLOPs. These results demonstrate that BCG-YOLO achieves a favorable balance between overall detection accuracy and computational efficiency, supporting the effectiveness of the proposed improvements for microscopic blood-cell detection.

From a clinical perspective, accurate blood cell detection is a prerequisite for automated hematological examination. Reliable localization and classification of red blood cells, white blood cells, and platelets are essential for downstream quantitative analyses, including cell counting, differential leukocyte analysis, and the identification of hematological abnormalities. Missed detection of platelets or inaccurate separation of overlapping cells may affect subsequent quantitative measurements and reduce the reliability of computer-assisted laboratory analysis. Therefore, improving the detection of small and densely distributed blood cells is of practical importance for enhancing the accuracy, consistency, and efficiency of routine hematological workflows.

The architectural design of BCG-YOLO was motivated by the practical challenges of blood-cell detection, including small target sizes, dense cellular distributions, partial overlap, and blurred boundaries. LGBNet increased mAP@0.5 from 91.7% to 93.1% while reducing computational complexity from 6.30 to 3.20 GFLOPs. This result indicates that the lightweight backbone effectively reduces redundant computation while preserving discriminative features, thereby improving the balance between detection accuracy and computational efficiency.

Dense distributions and partial overlap are common in peripheral blood-smear images and require effective integration of local features at different spatial scales. PSConv introduces poly-scale sampling at selected positions in the neck, enabling the model to capture complementary contextual and boundary information. The improvement observed after incorporating PSConv suggests that multi-scale local feature modeling contributes positively to the detection of blood cells in complex microscopic scenes.

DyHead further improves feature aggregation by adaptively recalibrating information across the scale, spatial, and task dimensions. The ablation results show that incorporating DyHead improves the overall detection performance, demonstrating the benefit of dynamic multi-level feature selection. Together with the qualitative examples of small and densely distributed blood cells, these results indicate that DyHead enhances the model's ability to represent targets with heterogeneous scales and spatial distributions.

The completed factorial ablation experiment evaluated all eight combinations of LGBNet, PSConv, and DyHead. The results provide systematic evidence that the three modules make complementary contributions to the overall performance of BCG-YOLO. In particular, LGBNet primarily improves computational efficiency, whereas PSConv and DyHead strengthen multi-scale feature fusion and adaptive feature representation. Their combination enables BCG-YOLO to achieve a favorable balance between detection accuracy and computational complexity.

Measured on the NVIDIA GeForce RTX 4090 under the same inference setup, BCG-YOLO processed 305.8 frames per second, compared with 297.6 FPS for YOLO11n. This measured throughput complements the reduction in computational cost from 6.30 to 3.80 GFLOPs and shows that the accuracy improvement was obtained without slower recorded inference. The computational savings are accompanied by increased weight storage. Relative to YOLO11n, BCG-YOLO requires an additional 4.56 MB in FP32 and 2.28 MB in FP16, corresponding to a 44.2% increase at either precision. This trade-off should be considered when deploying the model on memory-constrained devices. Weight storage alone does not determine peak inference memory, which also depends on intermediate activations, input buffers, and runtime implementation. Although the RTX 4090 measurements demonstrate favorable throughput under the tested conditions, memory consumption and inference performance on edge devices require evaluation on the target hardware.

All compared models were evaluated under a unified training protocol to provide a controlled comparison. This setting reduces variations caused by different data partitions and training procedures, although model-specific optimization may further improve the performance of individual architectures. The factorial ablation experiments were also conducted under the same protocol, thereby supporting a consistent comparison of the proposed modules. The five matched-seed experiment reported in Table 6 further showed that BCG-YOLO remained superior to YOLO11n across training randomizations under the fixed data split.

The paired bootstrap results provide additional support for the improvements in mAP@0.5 and mAP@0.5:0.95, as the confidence intervals for both paired differences excluded zero. Although the recall point estimate also favored BCG-YOLO, the confidence interval for its paired difference included zero, indicating that the evidence for a recall improvement was less conclusive on the original test set. These intervals characterize test-image sampling uncertainty for the selected model checkpoints and complement the repeated-seed and five-fold evaluations.

Despite these findings, the present study remains an internal proof-of-concept evaluation on BCCD. The dataset contains only 364 images and does not establish the range of variability encountered across laboratories, slide-preparation procedures, staining protocols, and imaging systems. Five-fold cross-validation and image-level bootstrap analysis strengthen the internal evaluation but do not replace independent external testing. Because no external validation was performed, robustness to different staining conditions and generalizability to other clinical settings remain unverified. The absence of independent external validation is therefore a major limitation. Future work should evaluate the model on independent multicenter datasets with compatible annotations for RBCs, WBCs, and platelets before drawing conclusions about clinical applicability.

## 6 Conclusions

This study proposed BCG-YOLO, an efficient blood-cell detection model that integrates LGBNet, PSConv, and DyHead into a YOLO11n-based framework. LGBNet reduces redundant computation while preserving effective feature representations, PSConv enhances multi-scale local feature fusion, and DyHead improves adaptive feature aggregation across the scale, spatial, and task dimensions. On the BCCD dataset, BCG-YOLO achieved an mAP@0.5 of 95.3%, an mAP@0.5:0.95 of 67.6%, and a recall of 89.8%, with a computational cost of 3.80 GFLOPs. The factorial ablation experiments covering all eight module combinations further demonstrated the complementary contributions of the proposed components and showed that the complete model achieved the best overall detection performance. The completed class-wise analysis showed improvements for RBCs, WBCs, and platelets, and the five matched-seed experiment confirmed consistent gains over YOLO11n under the fixed data split. These results support BCG-YOLO as a computationally efficient approach to microscopic blood-cell detection within the BCCD evaluation setting. Independent external and multicenter validation is required before its generalizability across staining conditions, imaging systems, and clinical settings can be established.

## Data Availability

The original contributions presented in the study are included in the article/Supplementary Material, further inquiries can be directed to the corresponding authors.
